# Drug interaction potential of Ankaferd blood stopper^®^ in human hepatocarcinoma cells

**DOI:** 10.55730/1300-0144.5605

**Published:** 2022-09-12

**Authors:** Aslı SEMİZ

**Affiliations:** Department of Biomedical Engineering, Faculty of Technology, Pamukkale University, Denizli, Turkey

**Keywords:** Ankaferd blood stopper^®^ (ABS), cytochrome P450 (CYP), HepG2 cells, drug-metabolizing enzymes, herb-drug interactions

## Abstract

**Background/aim:**

Ankaferd blood stopper^®^ (ABS) is an herbal extract consisting of mixtures of *Alpinia officinarum*, *Gycyrrhiza glabra*, *Vitis vinifera*, *Thymus vulgaris*, and *Urtica dioica* plants and has been used in recent years in Turkish medicine as a hemostatic agent. Despite its extensive usage, there is no information available about the drug interaction in HepG2 cells. The current work evaluated the effect of ABS on the expression of CYP1A1-1A2, CYP2E1, and CYP3A4 isozymes that are primarily involved in drug and carcinogen metabolism.

**Materials and methods:**

We selected HepG2 cells as in vitro cellular models of the human liver. The cells were treated with different concentrations of ABS [0.25%–40% (v/v)]. A crystal violet staining assay was used to determine the cytotoxicity of ABS. We examined drug-metabolizing enzymes, including 7-ethoxyresorufin O-deethylase (CYP1A1), 7-methoxyresorufin O-demethylase (CYP1A2), aniline 4-hydroxylase (CYP2E1), and erythromycin N-demethylase (CYP3A4), in vitro in HepG2 cells. The expression (mRNA, protein) levels of drug-metabolizing enzymes were analyzed by qPCR and Western blotting, respectively.

**Results:**

The EC05 and EC10 values for ABS were 0.37% and 0.52% (v/v), respectively. Therefore, 0.37% and 0.52% (v/v) doses were used for the remaining portion of this study. Investigation of the expression and activity levels revealed that CYP1A1-1A2, CYP2E1, and CYP3A4 activities were not affected by ABS significantly, with qPCR and Western blot results corroborating this result.

**Conclusion:**

Our study found that the activity, mRNA, and protein expression levels of CYP isozymes did not change with the application of ABS, suggesting that when humans are exposed to ABS, there may not be any risk associated with clinical drug toxicity, cancer formation, and drug metabolism disorders in humans.

## 1. Introduction

In the last decades, plant-based complementary or alternative treatments have received growing attention in the Western world. Because products derived from plants are natural, they are considered safe. However, it has been reported that herbs and herbal products may cause herb-drug interactions [1–4]. For instance, a range of plant and plant-based complementary therapeutics, including *Cyclamen trochopteranthum* [2], *Epilobium hirsutum* [3], *Thonningia sanguinea* [5], *Urtica urens* [6], ellagic acid [7], and hydroxycinnamic acid [4], can have pharmacokinetic drug interactions with the activity and expression of drug-metabolizing enzymes, in particular, cytochrome P450s.

Cytochrome P450s (CYPs) play a critical role in the metabolic bioactivation or detoxification of pharmaceutics as well as endogenous compounds, dietary components, and environmental xenobiotics [8]. The P450 superfamily shows broad substrate specificities toward both exogenous and endogenous components. Particularly, CYP1, CYP2, and CYP3 gene families are responsible for the metabolism of over 90% of all drugs and xenobiotics [9]. Among these CYPs, CYP1A, 2E, and 3A have received growing attention due to their ability to metabolize a large variety of drugs and their role in the metabolism of carcinogens [2,4,6,10,11].

CYP1A and 2E subfamilies are primarily involved in the metabolism of carcinogens, mutagens and environmental pollutants [12,13]. CYP1A isoform plays a critical role in metabolizing endogenous and exogenous substrates including aromatic amines and heterocyclic compounds [9]. In particular, CYP1A isoforms play a role in carcinogenic formations such as lung, colorectal, and breast cancer [14,15]. Moreover, it has been shown that compounds isolated from some dietary/nondietary plants have significant suppressive effects on various types of cancer by inhibiting the expression or activities of CYP1A isoforms [16]. CYP2E1 is important in carcinogen metabolism. It can metabolize and activate drugs and hydrophobic substrates, such as ethanol, chloroform, benzene, nitrosamines, and carbon tetrachloride [9]. In addition, various phytochemicals antagonize the carcinogenic and mutagenic effects of nitrosamines by inhibiting CYP2E1 activity or decreasing protein levels [17,18]. CYP3A4 is another important CYP isoform as it is responsible for the biotransformation of approximately 50% of all pharmaceuticals (such as calcium channel blockers, antibiotics, antiviral drugs, anti-histamines and HMG-CoA reductase inhibitors) [9,19]. CYP3A4 also plays a significant role in the biotransformation and biosynthesis of endogenous substances, such as cholesterol, steroids, vitamins, and other lipids [9]. Furthermore, various herbal extracts modulate CYP3A4 activity and these plant components interact with the enzyme [20,21]. Consequently, it is critical to monitor drug toxicity with herbs and herbal medicines used as alternative therapies that are moderate-to-strong inhibitors and/or activators of the CYPs.

Ankaferd blood stopper^®^ (ABS) is a prominent hemostatic agent that is commonly used in Turkey [22,23]. ABS is a standard mixture of *Alpinia officinarum*, *Gycyrrhiza glabra*, *Vitis vinifera*, *Thymus vulgaris*, and *Urtica dioica* [23–26]. The herbs found in ABS can affect angiogenesis, blood cells, endothelial cells, and cellular proliferation [22,24,25,27–29]. Additionally, ABS is known to have antifungal, antiinflammatory, antioxidant, and antibacterial activities [30–32]. It has been shown that *T. vulgaris* leaves have antioxidant effects comparable to potent antioxidant compounds such as butylated hydroxytoluene and α-tocopherol [24]. *G. glabra* root has antiangiogenic, antiinflammatory, antithrombotic, antioxidant, and antiatherosclerotic effects [27,33,34]. Likewise, many studies have reported that *A. officinarum*, *V. vinifera*, and *U. dioica* possess various biological and pharmacological activities like antiproliferative, antioxidant, antiinflammatory, antiallergic, antifungal, antibacterial, and antiviral effects [35–37].

As a local hemostatic agent, ABS creates this effect by inducing a protein network formation between blood proteins and ABS [22,32]. ABS has been also successfully used in abdominal, thoracic, pelvic, and dental surgeries [38–40]. Additionally, it has become known to stimulate the regeneration of bone, muscle, intestinal tract, and urogenital tract mucosae after surgical procedures [40–42]. Despite its wide usage, there is no information available on the effect of ABS on drug metabolism and the potential for drug interactions. Therefore, the current study aims to identify the effect of ABS on drug-metabolizing CYPs.

## 2. Materials and methods

### 2.1. Cell culture

HepG2 [human liver cancer cell line, European Collection of Cell Cultures (ECACC)] cells were routinely maintained in DMEM (10% FBS) supplemented with 1% penicillin/streptomycin at 37 °C in a humidified incubator with 5% CO_2_, subcultured twice per week.

### 2.2. Cell viability assay

HepG2 cells were seeded in 96-well culture plates at a density of 1 × 10^3^ per well and incubated overnight in DMEM before ABS treatment. HepG2 cells were exposed to different concentrations of ABS [0.25%–40% (v/v)] for 24 h. The same amount of fresh medium without ABS was used as a negative control (untreated cells). After incubation, the medium was discarded and attached cells were stained with an ethanolic solution of crystal violet [0.5% (w/v)] for 10 min. After incubation, plates were washed with distilled water and adsorbed dye was desorbed with 0.1 M sodium-citrate (pH 4.2). Cell viability was evaluated by measuring the optical density at 630 nm, and expressed as the percentage of viable cells compared to the negative control.

### 2.3. Cell homogenate preparation and enzyme assays

HepG2 cells were seeded in flasks (1 × 10^6^ cells) and exposed to 0.37% and 0.52% (v/v) ABS doses. After 24 h, the cells were harvested with lysis buffer (0.1 M phosphate buffer pH 7.8, EDTA 2 mM, 0.2% Triton X-100, ɛ-ACA 0.3 mM, DTT 1 mM, APMFS 0.5 mM). The total protein of the cells was determined by the bicinchoninic acid protein assay using protein standard (bovine serum albumin).

The catalytic activity of CYP2E1 of negative control and ABS-treated cells was determined by aniline hydroxylation to p-aminophenol [43]. The catalytic activities of CYP1A1 and 1A2 towards 7-ethoxyresorufin (ER) and 7-methoxyresorufin (MR), respectively, were measured spectrofluorometrically according to the procedure outlined by Burke and Mayer [44] as described by Sen and Arinc [45]. CYP3A4 catalytic activity of negative control and ABS-treated cells was determined by N-demethylation of erythromycin using the Nash method [46].

### 2.4. Gel electrophoresis and Western blotting

Protein separation and Western blot were carried out as described by Sen and Arinc [45]. Briefly, proteins were separated on 12% polyacrylamide gel using the discontinuous Laemmli system [47]. After electrophoretic separation, the proteins from the gel were electrophoretically transferred onto a membrane (nitrocellulose) for 90 min at 90 V at 4 °C using a Transblot electrophoretic transfer cell system (Bio-Rad). After the transfer, membrane surfaces were blocked by 5% nonfat dry milk prepared in TBST buffer [Tris-HCl 20 mM, NaCl 400 mM, pH 7.4, and 0.1% Tween-20] for 1 h at room temperature and incubated at 4 °C overnight with 1:200 dilutions of polyclonal antihuman CYP1A2, CYP2E1, and CYP3A4 antibodies. The membranes were washed with TBST three times (5 min), and then incubated at room temperature for 2 h with HRP-labeled secondary antibodies in TBST (1:5000) with nonfat dry milk (5%). After washing with TBST (3 × 5 min), protein detection was performed with SuperSignal^®^ West Pico Chemoluminescent Substrate (Pierce, Rockford, IL, USA), followed by imaging through Odyssey^®^ Fc Imaging System (LI-COR). Densitometric quantification of the bands was performed using the Image Studio™ Lite Quantification Software. All CYP isoforms were normalized to β-actin.

### 2.5. RNA isolation and cDNA synthesis

The RNA samples were extracted from cells using RNeasy Plus Universal Kit (Qiagen, Valencia, CA, USA), as described in the manufacturer’s instructions and as optimized in our laboratory. The 260/280 ratio was used for determining RNA concentration and purity. Subsequently, 2 μg of total RNA was reverse transcribed using Easy Script cDNA Synthesis Kit (ABM).

### 2.6. Quantitative real-time PCR (qPCR)

The RNA expression levels of CYP1A1, 1A2, 2E1, and 3A4 genes in HepG2 cells were measured with SYBR Green qPCR Master Mix (GM, Taiwan). The gene-specific primer sequences are shown in Table 1. Changes in mRNA expression of the different genes were normalized with an internal standard β-actin mRNA. Relative expression of mRNA was quantified by the 2^−ΔΔCt^ method [48]. Each experiment was carried out in triplicate and each was repeated three times.

### 2.7. Statistical analysis

The data were analyzed by using the Student’s t-test, and a *p*-value below 0.05 was selected as the statistical significance level. The analyses were performed using Minitab 13 (State College, PA, USA) statistical analysis software. All data are shown as the mean values ± standard error of triplicate analyses.

## 3. Results

### 3.1. Effect of the ABS on HepG2 cells

**Figure 1:**
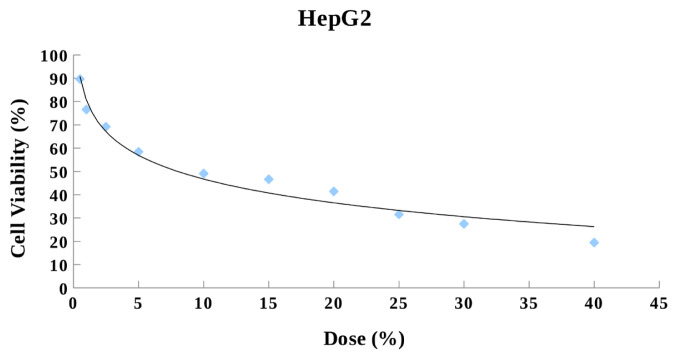
Effect of ABS on cellular viability of HepG2 cells. The cells were treated with increasing concentrations of ABS for 24 h before cell viability assay. All data are shown as the mean values ± standard error of triplicate analyses.

The effect of ABS on HepG2 cell survival was determined by crystal violet staining. The nontoxic doses of ABS on HepG2 cells were determined (Figure 1). The EC05 and EC10 values for ABS were calculated as 0.37% and 0.52% (v/v), respectively. Therefore, 0.37% and 0.52% (v/v) doses were used in this study.

### 3.2. Effect of the ABS on P450 activities

HepG2 cells were harvested following a 24-h treatment with ABS, and cell lysates were used as a source of enzyme. The effect of ABS on the CYP450 enzyme activities was determined with a spectrophotometer using suitable probe substrates (Table 2). EROD (associated with CYP1A1) and MROD (associated with CYP1A2) activities were not altered significantly in ABS-treated HepG2 cells when compared to the untreated control cells. The effect of ABS on A4H (associated with CYP2E1) and ERND (associated with CYP3A4) activities are given in Table 2. Administration of ABS did not alter A4H and ERND activities relative to the control cells (Table 2).

### 3.3. Effect of the ABS on P450 proteins and mRNA levels

**Figure 2:**
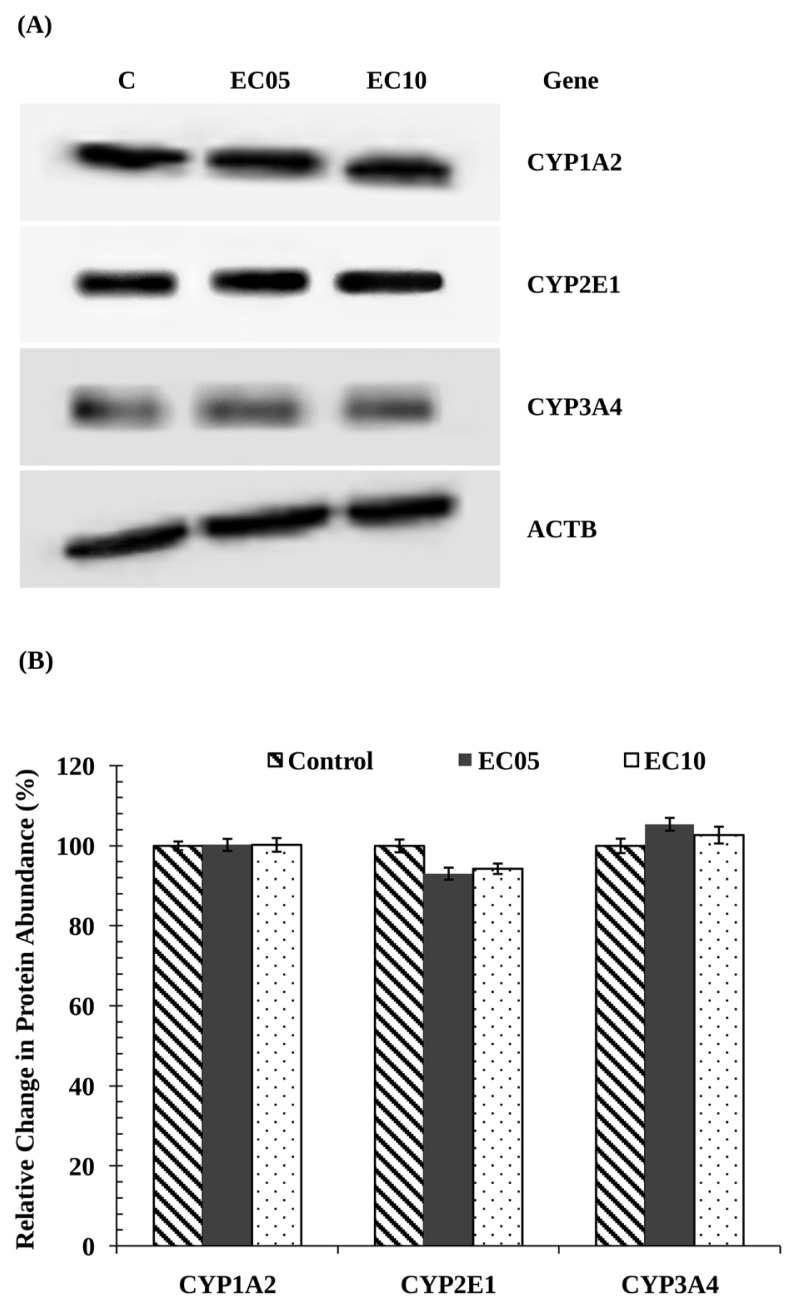
**(A)** The expression levels of CYP1A2, 2E1, and 3A4 proteins in control and ABS-treated HepG2 cells. The materials and methods section explains the treatments that have been carried out. All lanes contained 80 μg of protein. **(B)** Graphical presentation of the semiquantitative data from Western blot. All data are shown as the mean values ± standard error of triplicate analyses. The relative control values were taken as 100%.

**Figure 3:**
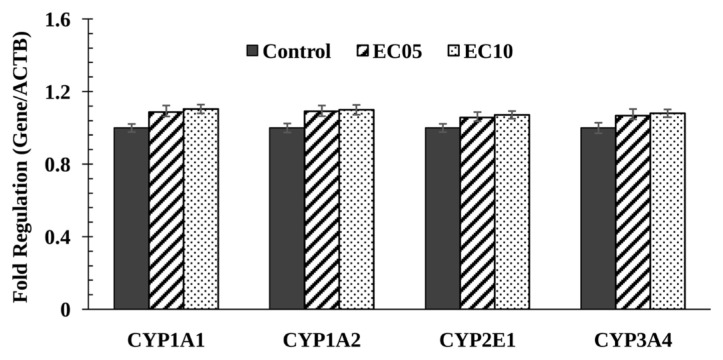
mRNA expression levels of CYP1A1, 1A2, 2E1, and 3A4 in control and ABS-treated HepG2 cells. The materials and methods section explains the treatments that have been carried out. Normalization of the qPCR data was carried out using internal control gene, namely β-actin. The bar chart represents the average fold changes calculated using the formula 2^−ΔΔCt^.

To investigate the changes in expression levels of CYP isoforms in ABS-treated HepG2 cells and untreated control cells, proteins were separated by size on polyacrylamide gels and then analyzed on immunoblots probed using specific antibodies recognizing CYP1A2, 2E1, and 3A4 isoforms (Figures 2a and 2b). Western blot analysis showed that CYP1A2, 2E1, and 3A4 protein levels were not altered in the ABS-treated HepG2 cells compared with the control cells (Figures 2a and 2b). The effect of ABS on the CYP1A1, 1A2, 2E1, and 3A4 mRNA expression was also analyzed using qPCR in this study. The relative expression levels of CYP1A1, 1A2, 2E1, and 3A4 did not vary between ABS-treated HepG2 cells and control cells (Figure 3).

## 4. Discussion

Herbal medicines as alternative and/or complementary therapy have long been used in Asia and the Western world for centuries. However, it has been noted that medicinal plants have been used due to traditional beliefs that can cause interactions between herbal and conventional medicines. Between these interactions, both the inhibition and induction of cytochrome P450 enzymes have been shown to be significant [1,21]. Herbal medicines can act as inhibitors or inducers of P450s, resulting in either enhancement of the therapeutic efficacy or treatment failure, respectively [49]. That is why herbal remedies are remarkable facts that can affect P450-mediated drug metabolism.

Functionally, CYP1A, 2E, and 3A families have received great interest in the last years due to their significant capacity in the metabolism of a large majority of drugs and their role in carcinogenesis [2,4,6,10,11]. In recent years many studies were devoted to evaluating the inhibitory activity of many plants on these enzyme families [5,21]. Ankaferd blood stopper^®^ (ABS) is a hemostatic drug of five different plant origins that has been developed in Anatolia and accepted in the universal literature [23,28]. ABS has been used to stop bleeding. Numerous case reports show that ABS is used safely and effectively in bleeding in rectal and peptic ulcers, cardiac surgery, extremity laceration, thyroidectomy, surgical and dental procedures, and adenoidectomy [23]. In addition to its hemostatic effect, ABS is known to have antiinflammatory, antioxidant, and antibacterial activities due to its components [30–32]. ABS also has a strong hepatoprotective feature due to its antioxidant and antiinflammatory properties [29,50].

The liquid chromatography-mass spectrometry (LC-MS) analysis of ABS revealed the presence of various antioxidant molecules, including apigenin, 3,4-divanillyltetrahydrofuran, galangin, tocotrienols, estriol, oenin, thymol, tryptophan, lycopene, tomatine, butylated hydroxyanisole, butylated hydroxytoluene, and tertiary butylhydroquinone [51]. Moreover, the concentrations of these compounds were not affected by the exposure to synthetic gastric fluid [51]. Most of these antioxidants have anticancer effects, and numerous studies demonstrated the proliferation-inhibiting capacity of ABS on cancer cells [52–55]. Furthermore, the inhibition of cellular reproduction and reduction in the viabilities of colon cancer cells (CaCo-2) were also observed after ABS addition [52]. Turk et al. also showed the growth inhibitory functions of ABS on melanoma cells [55]. In another study by Kocyigit et al., it was revealed that ABS induces apoptosis and cytotoxicity in melanoma cells [54]. The antiproliferative effects of ABS on lymphoid neoplastic cell lines were demonstrated in a study by Akalın et al. [53].

The present study is the first attempt to investigate the effects of ABS on drug metabolism in HepG2 cells. Human hepatoblastoma-derived cell line HepG2 was developed with the intention that would be a suitable hepatic model in early drug and toxicology screening [56]. HepG2 cell line exhibits genotypic and phenotypic characteristics of hepatic cells [57]. Furthermore, HepG2 cells express functional biotransformation enzymes involved in xenobiotic pathways. Therefore, HepG2 cells were used throughout this study.

Among all CYP isoforms, CYP1A has a high priority because of its importance in the metabolism of mutagenic/carcinogenic xenobiotics [7]. CYP1A isoform is the major P450 enzyme that metabolizes carcinogens into highly reactive epoxides. There are studies in the literature showing that this isoform is involved in the metabolic bioactivation of aromatic and heterocyclic amines, benzo(a)pyrene, and polyaromatic hydrocarbons [12,13]. Considering that CYP1A2 catalyzes approximately 10% of known drugs, it should be taken into account that taking these herbal products together with antidepressant group drugs may lead to significant drug interactions, leading to overdose and dangerous situations. In this study, EROD and MROD activities (associated with CYP1A1-CYP1A2) were not altered significantly in ABS-treated HepG2 cells as compared to the untreated control cells (Table 2). Besides, the expression of CYP1A1-1A2 protein and mRNA were unchanged by ABS treatment (Figures 2a, 2b, and 3). The CYP3A subfamily comprises the largest portion of CYP450 proteins in the liver of adults [58] and is included in the metabolism of over half of the therapeutics such as sedatives, antibiotics, calcium channel blockers, antihistamines, statins, and anticancer agents [8]. For this reason, changes in the CYP3A4 isoform as a result of ABS administration may cause disturbances in drug metabolism and clinical drug toxicity. However, in our study, ABS treatment did not change CYP3A4-associated ERND activity significantly nor CYP3A4 mRNA and protein levels as compared to the untreated control cells (Table 2; Figures 2a, 2b, and 3). CYP2E1 plays an important role in toxicology and carcinogenesis as well as drug metabolism [12]. There are studies in the literature showing that CYP2E1 converts many xenobiotics such as nitrosamines, carbon tetrachloride, and benzene into mutagenic, electrophilic, and tumor-promoting metabolites [12,59]. In this study, ABS treatment did not change CYP2E1-associated A4H activity significantly nor CYP2E1 mRNA and protein levels as compared to the untreated control cells (Table 2; Figures 2a, 2b, and 3). As a result, people exposed to ABS do not have the risk of cancer formation and liver toxicity from chemicals metabolized by CYP2E1.

In the present study, the effect of ABS on P450 isozymes (CYP1A1-1A2, 2E1, and 3A4), which are associated with the biotransformation of many xenobiotics, such as various pharmaceuticals and carcinogenic agents, was determined. The effects of ABS on phase I enzymes (CYP1A1-1A2, 2E1, and 3A4) in HepG2 cells were reported for the first time. CYPs gene expressions are regulated by the activation of nuclear receptors, including the aryl hydrocarbon receptor (AhR), the constitutive androstane receptor (CAR), and the pregnane X receptor (PXR); all these can be activated by xenobiotics or ligands [9,60]. ABS administration did not lead to any significant changes in CYP1A1-1A2, 2E1, and 3A4 activity and protein and mRNA levels in HepG2 cells, suggesting that ABS did not affect transcriptional regulators, including AhR, CAR, and PXR.

In conclusion, because the activity, protein, and mRNA levels of cytochrome P450 isozymes do not change with ABS application, we can say that individuals exposed to ABS may not have a risk of impaired drug metabolism, clinical drug toxicity, and cancer formation.

## Figures and Tables

**Table 1:** Gene-specific primer sequences.

Gene name	Primer Sequence (5′->3′)	TM (°C)
*hCYP1A1*	F→ AGC GGA AGT GTA TCG GTG AGA R→ CTG AAT TCC ACC CGT TGC A	55
*hCYP1A2*	F→ ACT TCG ACC CTT ACA ATC AG R→ CAC TGT TCT TGT CAA AGT CC	53.3
*hCYP2E1*	F→ GCATCT CTT GCC TAT CCT T R→ ATG GAC CTA CCT GGA AGG ACA T	61
*hCYP3A4*	F→ GCC TGG TGC TCC TCT ATC TA R→ GGC TGT TGA CCA TCA TAA AAG	54.7
*hβACTIN*	F→ TCC TCC TGA GCG CAA GTA CTC R→ CTG CTT GCT GAT CCA CAT CTG	59

**Table 2:** The effect of ABS on CYP450 enzyme activities in HepG2 cells.

Enzyme activities	Control	EC05: 0.37%	EC10: 0.52%
EROD (pmol Resorufin/min/mg prot.)	5.52 ± 0.23	5.44 ± 0.12	5.48 ± 0.14
MROD (pmol Resorufin/min/mg prot.)	6.88 ± 0.53	6.82 ± 0.57	6.87 ± 0.47
A4H (nmol HCHO/min/mg prot.)	0.087 ± 0.028	0.084 ± 0.01	0.082 ± 0.011
ERND (nmol HCHO/min/mg prot.)	0.61 ± 0.088	0.55 ± 0.06	0.56 ± 0.063

All data are shown as the mean values ± standard error of triplicate analyses.
